# SARSMutOnto: An Ontology for SARS-CoV-2 Lineages and Mutations

**DOI:** 10.3390/v15020505

**Published:** 2023-02-11

**Authors:** Jamal Bakkas, Mohamed Hanine, Abderrahman Chekry, Said Gounane, Isabel de la Torre Díez, Vivian Lipari, Nohora Milena Martínez López, Imran Ashraf

**Affiliations:** 1 LAPSSII Laboratory, Graduate School of Technology, Cadi Ayyad University, Safi 46000, Morocco; 2 Department of Telecommunications, Networks, and Informatics, LTI Laboratory, ENSA, Chouaib Doukkali University, Eljadida 24000, Morocco; 3 MIMSC Laboratory, Graduate School of Technology, Cadi Ayyad University, Essaouira 44000, Morocco; 4 Department of Signal Theory and Communications and Telematic Engineering, University of Valladolid, Paseo de Belén, 15, 47011 Valladolid, Spain; 5 Research Group on Foods, Nutritional Biochemistry and Health, Universidad Europea del Atlántico, Isabel Torres 21, 39011 Santander, Spain; 6 Department of Project Management, Universidad Internacional Iberoamericana Campeche, Mexico City 24560, Mexico; 7 Fundación Universitaria Internacional de Colombia Bogotá, Bogotá 11001, Colombia; 8 Research Group on Foods, Nutritional Biochemistry and Health Universidad Internacional Iberoamericana, Arecibo, PR 00613, USA; 9 Research Group on Foods, Nutritional Biochemistry and Health Universidade Internacional do Cuanza, Cuito EN250, Angola; 10 Department of Information and Communication Engineering, Yeungnam University, Gyeongsan 38541, Republic of Korea

**Keywords:** ontology, genome structure, SARS-CoV-2, mutation, lineage

## Abstract

Mutations allow viruses to continuously evolve by changing their genetic code to adapt to the hosts they infect. It is an adaptive and evolutionary mechanism that helps viruses acquire characteristics favoring their survival and propagation. The COVID-19 pandemic declared by the WHO in March 2020 is caused by the SARS-CoV-2 virus. The non-stop adaptive mutations of this virus and the emergence of several variants over time with characteristics favoring their spread constitute one of the biggest obstacles that researchers face in controlling this pandemic. Understanding the mutation mechanism allows for the adoption of anticipatory measures and the proposal of strategies to control its propagation. In this study, we focus on the mutations of this virus, and we propose the SARSMutOnto ontology to model SARS-CoV-2 mutations reported by Pango researchers. A detailed description is given for each mutation. The genes where the mutations occur and the genomic structure of this virus are also included. The sub-lineages and the recombinant sub-lineages resulting from these mutations are additionally represented while maintaining their hierarchy. We developed a Python-based tool to automatically generate this ontology from various published Pango source files. At the end of this paper, we provide some examples of SPARQL queries that can be used to exploit this ontology. SARSMutOnto might become a ‘wet bench’ machine learning tool for predicting likely future mutations based on previous mutations.

## 1. Introduction

Severe acute respiratory syndrome coronavirus (SARS-CoV) evolved from China in 2002, and Middle East respiratory syndrome coronavirus (MERS-CoV) originated in the Middle East in 2012  [1]. They are two coronavirus-type viruses of zoonotic origin that have already sounded the alarm about the dangers that this kind of virus can generate. Unlike its predecessors from the same coronavirus family, the severe acute respiratory syndrome coronavirus 2 (SARS-CoV-2), which was discovered at the end of 2019 in Wuhan, China   [2,3], has spread across the world with exceptional speed. The COVID-19 disease caused by the SARS-CoV-2 virus, officially declared as a pandemic by the World Health Organization (WHO) in March 2020, continues to spread and affect individuals of all ages around the world. As of 15 January 2023, the WHO had reported more than 661 million confirmed cases, including 6.7 million deaths, with incalculable health, social, and economic costs  [4]. Several vaccines were developed in record time. Massive vaccination campaigns have been launched, resulting in a significant reduction in mortality and hospitalization rates, especially among the elderly  [5]. Nevertheless, the virus is still present and permanently evolving, so there is a need for any method, computational technique, or new tool that can be used to provide information about its evolution. A variety of research projects have been triggered related to this pandemic and all related topics, to understand it, predict its spread, and propose measures to besiege it. Bioinformatics and ontology engineering research projects have been contributing to this movement by proposing many ontologies to model domains related to the pandemic. Most of these ontologies are available through the National Center for Biomedical Ontology (NCBO BioPortal)  [6]. Among these ontologies, we cite the Gene Ontology (GO)  [7] proposed in 2000 and extended over the years. Its latest release (September 2022) contains 50,977 classes. This ontology describes the knowledge of the biological domain according to three aspects: molecular function, cellular component, and biological process. GO has a general purpose dealing with the management of information regarding genes and gene products amongst different species. The ontology provides a description of the genes, their relationships, and functions. After the success of GO, several ontologies have emerged over the past years. Disease Ontology (DO) is one of them, first appearing in 2012  [8]. It is considered the core for human disease semantic integration by providing a standardized ontology for describing disease terms. Its latest release (7 January 2022) describes the complexity of more than 10,000 human diseases. Having extensible content allowing for the knowledge sharing of new discoveries, DO has been at the origin of several other new ontologies and projects. Another important and specific ontology is the Infectious Disease Ontology (IDO)  [9] which is designed as a set of interoperable ontologies covering the infectious diseases domain. These ontologies are built around the core ontology (IDO-Core), which provides a set of relevant entities to describe the clinical and biomedical aspects of infectious diseases. Since COVID-19 is an infectious disease caused by the coronavirus, the Coronavirus Infectious Disease Ontology (CIDO)  [10] appeared as an extension of IDO and covers everything that depends on infection with the different coronaviruses and associated diseases. A more specific ontology called IDO-COVID-19 (COVID-19 Infectious Disease Ontology)  [11] extends CIDO by describing the domain of infections with SARS-CoV-2 virus strains and related COVID-19 disease. Continuing with the COVID-19 disease domain, we cite the COVID-19 ontology  [12] with 2270 classes for describing molecular and cellular entities and their roles in virus–host interactions and the virus life cycle, as well as a wide range of medical and epidemiological concepts related to COVID-19. Finally, we cite the OntoRepliCov ontology  [13] that describes the genomic structure of the SARS-CoV-2 virus and the different steps of its replication process.

After a survey of existing ontologies, we noted that, so far, only the last CIDO ontology update included a limited number of terms for GISAID clades, Pango lineages, and WHO variants. It provides about 39 specific classes that describe specific SARS-CoV-2 variants  [14]. However, we have not found any ontology describing all Pango lineages and mutations. Information about mutations and lineages is available and accessible on the internet, but scientists sometimes need more complex information that is not explicitly available. The SARS-Cov-2 variant accumulates mutations to produce new lineages. Data on these mutations and involved genes are tracked and reported by outbreak.info  [15]. In this work, we retrieve these data and additional data from other sources and restructure them into an ontology. Called SARSMutOnto, this ontology provides a detailed description of the mutations and lineages reported by Pango scientists and researchers  [16]. The SARSMutOnto ontology can be found on the bioportal at https://bioportal.bioontology.org/ontologies/SARSMUTONTO and on GitHub at https://raw.githubusercontent.com/jbakkas/SARSMutOnto/main/SARSMutOnto.owl.

## 2. Background

### 2.1. SARS-CoV-2 Virus Structure

**Figure 1:**
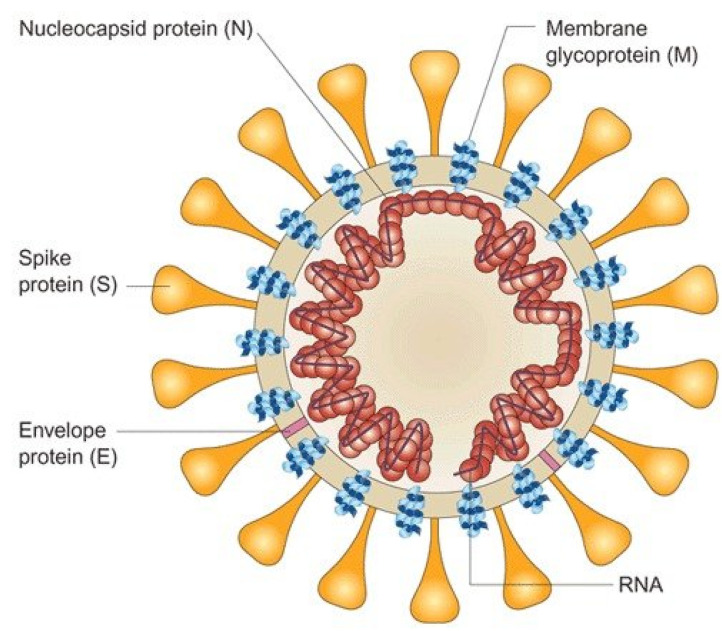
General structure of SARS-CoV [19].

**Figure 2:**
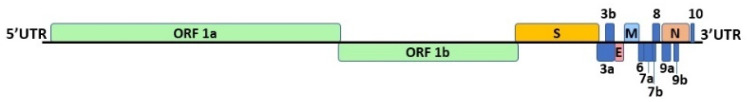
The set of genes making up the SARS-CoV 2 genome.

First time occurring, SARS-CoV-2 virus has been classified in the coronavirus family. Its genome structure corresponds to the specific genetic characteristics recognized for Coronaviruses. As detailed in  [2,17,18], this genome is composed of two replicate proteins ORF1a, ORF1b, and four structural proteins: the spike protein (S), the envelope protein (E), the nucleocapsid proteins (N), and the membrane glycoprotein (M). Between these proteins, nine other proteins are distributed which are called accessory proteins ORF3a, ORF3b, ORF6, ORF7a, ORF7b, ORF8, ORF9a, ORF9b, and ORF10. Figure 1 and Figure 2 show the sequence of the genes from the 5’-UTR end to the 3’-UTR end.

### 2.2. Mutation, Lineage, and Recombinant Virus

The SARS-CoV-2 virus, like any other virus, has mutated continuously since its emergence. A viral mutation is a change in the virus genome during the virus multiplication process. This change may have no impact and thus produce a neutral mutation, or it may have enough impact to develop another strain of the virus with different properties, or to a lesser extent to evolve a lineage. Lineages are related viruses descended from common ancestors. Most lineages disperse naturally. Natural selection always favors viral lineages that have acquired characteristics that allow them to survive more easily. Some mutations give the virus a certain evolutionary advantage, which may make it more contagious, fiercer, or more resistant to the immune system and vaccines. A lineage with such mutations becomes more contagious than others, and therefore more dominant. When several variants infect the same cell and, during multiplication processes, a hybrid genome results from portions of several genomes, then a so-called ‘recombinant’ virus is produced. Viral recombination is favored if there is a large circulation of virus variants.

### 2.3. Lineage Nomenclatures

Because of its wide spread, SARS-CoV-2 mutations have given rise to thousands of lineages from which several variants have emerged worldwide, with different characteristics from one variant to another. In early 2021, The most popular variants were the British variant  [20], the Indian variant  [21], the South African variant  [22], and the Brazilian variant  [23]. In the media and among the general public, the variants were usually designated by their original country name. The WHO has renamed the most widespread variants with Greek letters  [24], to have names that are easy to remember, but also to avoid the stigma of names designating the countries in which the variant first appeared. For example, the WHO gave the name ‘Alpha’ to the variant known in the media as the ‘British variant’, and ‘Beta’ to the variant known as ‘South Africa’, etc. In reality, the scientists assign names not only to variants but also to all reported lineages. The main nomenclatures available are those proposed by Pango, GISAID  [25], and Nextstrain. While the GISAID  [26] and Nextstrain  [27] nomenclatures provide an overview of clade trends; the Pango nomenclatures offer detailed information on lineages, allowing early prediction of local lineage expansion. For example, Pango assigned ‘B.1.1.7’ to the ‘Alpha’ variant, while GISAID used ‘GR/501Y.V1’, and Nextstrain used the name ‘20I/S:501Y.V1’. Pango researchers propose a lineage naming algorithm and the Pangolin lineage naming tool that dynamically attributes names to lineages; the latter being available both as a web application and as a command line tool  [28,29].

### 2.4. SARS-CoV-2 Mutations

During the replication process, new mutations may occur anywhere in any gene composing the virus genome. The mutation becomes interesting when it confers additional characteristics to this virus. Most researchers have linked the SARS-CoV-2 propagation speed to mutations detected in the spike protein (S) gene. Indeed, the virus relies on this protein and more specifically on the receptor-binding domain (RBD) of this protein to bind to lung cell surface receptors (ACE2) when entering the host cell  [30,31]. The S protein is the main target of antibodies generated either by the natural reaction of an infected human body or by vaccination. Mutations occurring in this area influenced the virus’ ability to enter cells by increasing or decreasing the efficiency of binding to the ACE2 receptor  [32,33]. Lineages resulting from these mutations develop characteristics that allow increased contagiousness and evasion of cellular immunity  [34]. Examples of these lineages include B.1.427/B.1.429  [35] assigned by the WHO with the Greek letter Epsilon. Other more well-known and dominant variants causing successive waves of the pandemic around the world include the Alpha, Beta, Gamma, and Delta variants. The fifth wave of the pandemic was triggered on 26 November 2021, when the WHO announced the appearance of the Omicron variant (B.1.1.529). In this variant, a very high number (32) of mutations are found in the spike protein S, compared to its devastating predecessor Delta, which has only five mutations.

Mutations in other genes, other than the spike protein, have also been the focus of study. One of these studies links mutations in the gene encoding ORF3a accessory protein to increased mortality rates  [36]. We noted that in order to study and understand the characteristics of a lineage, and to predict the behavior of future variants, it is necessary to study the mutations they have undergone, especially recurrent mutations. Through this work, we present an ontology that gathers all mutations reported by researchers in great detail since the first strain of the virus was found.

## 3. Materials and Methods

**Figure 3:**
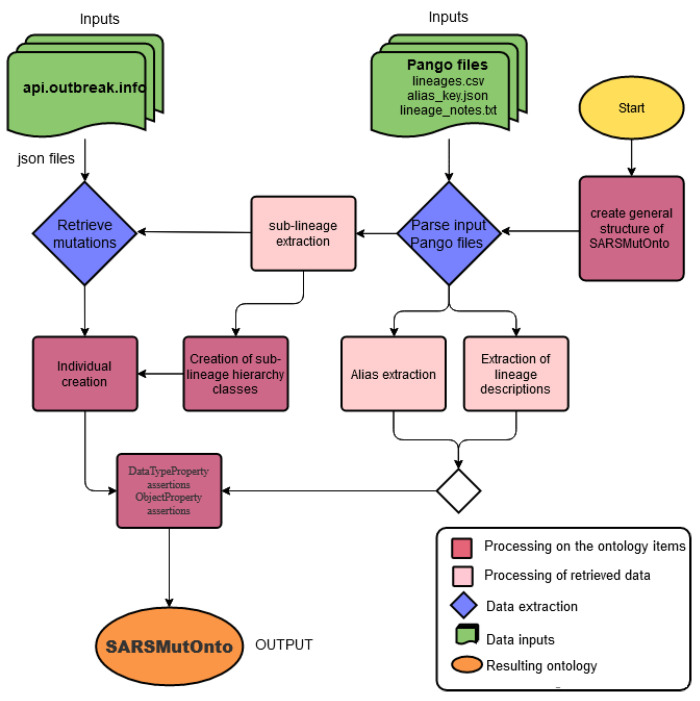
Workflow diagram of the steps followed in this study.

In this study, we present a lightweight ontology specifically designed to describe lineages and associated mutations in detail. Since the SARS-CoV-2 virus continues to evolve, the study also provides a tool to automatically regenerate updated versions. Our data sources are mainly Pango files and outbreak.info API  [15]. The workflow diagram of the approach followed in this study is shown in Figure 3.

**Figure 4:**
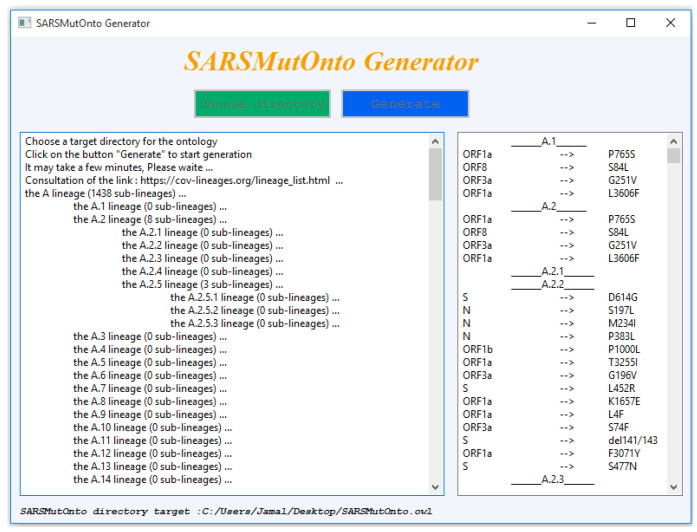
SARSMutOnto Generator: tool used to generate SARSMutOnto.

New lineages are identified around the world every day and are reported regularly by Pango. To keep our ontology up to date, we have developed the SARSMutOnto generator tool shown in Figure 4. This tool allows the automatic generation of the updated release of SARSMutOnto.

The entries of this system, as shown in the workflow diagram in Figure 3, are of two types. The Pango files and the API are provided by outbreak.info. The files are parsed to extract information about the lineages and their hierarchy. The information includes Pango-assigned lineage name, direct ancestor name or ancestor names if a recombinant, WHO-assigned name, lineage description, and alias; the aliases are retrieved using the ‘pango_aliasor’ Python library available via the following link: ‘https://github.com/corneliusroemer/pango_aliasor’. The API is consulted to retrieve the details of the mutations for each lineage. The API provides the mutation and the gene where it occurred.

**Figure 5:**
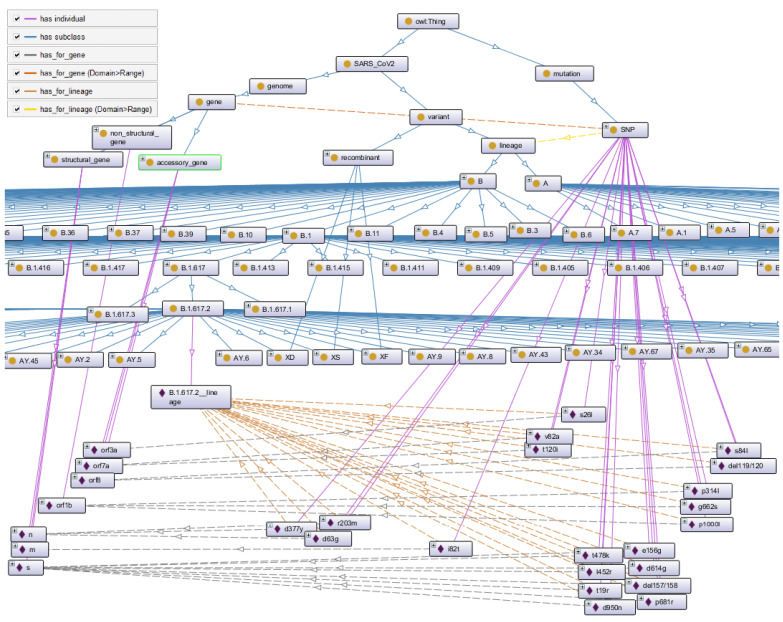
Portion of the ontology that describes the Delta (B.1.617.2) variant.

The generation process starts with the creation of the ontology’s general structure. This structure is composed of the following classes: ‘SARS-CoV-2’, ‘variant’, ‘lineage’, ‘recombinant’, ‘genome’, ‘gene’, ‘structural_gene’, ‘non_structural_gene’, ‘accessory_gene’, ‘mutation’, and ‘SNP’. Then, we create the individuals that represent the fifteen genes comprising the genome. These components are linked to each other by inheritance relationships and by object properties as shown in Figure 5. The retrieved entries are then used to generate classes, individuals representing lineages, and mutations to the ontology using the Owlready2  [37] Python package dedicated to ontology-oriented programming.

The steps followed by the SARSMutOnto Generator tool to generate the ontology are shown in Algorithm 1 using the pseudo-code. The graphical interface of the tool is provided in Figure 4. It is divided into two sections; the first one on the left displays the progress of the lineages, and the second one on the right displays the mutations extracted from the outbreak.info API for each lineage. It is developed using the 3.10.0 Python version. The tool’s source code is open and available on GitHub via the following link: https://github.com/jbakkas/SMOGenerator.
**Algorithm 1:** Ontology generation algorithm**Inputs:** Pango fils and outbreak.info API1:Ontology initialization2:Retrieve lineage list from lineage.yml3:Retrieve alias from alias_key.json using pango_aliasor4:Retrieve descriptions from lineage_notes.txt5:**for** Each lineage **do**6:   Create corresponding ontology class7:   Connect the class to its direct ancestor(s)8:   if a recombinant lineage connect to ‘recombinant’ class by ‘is_a’ link9:   Create individual from class10:   Add alias, if exists, by assertion of ‘has_for_description’ ataTypeProperty11:   Add description by assertion of ‘has_for_description’ ataTypeProperty12:   Extract list of lineage mutations and their genes13:   **for** Each mutation **do**14:     Create an individual of ’SNP’ class15:     Connect mutation to gene by assertion of ‘has_for_gene’ ObjectProperty16:     Connect mutation to lineage by assertion of ‘has_for_lineage’ ObjectProperty17:   **end for**18:**end for****Output:** SARSMutOnto ontology

## 4. Results

### 4.1. The Proposed Ontology

Biomedical ontologies, and especially those that extend the “GO“ ontology, generally describe biological domains in three aspects; cellular component, molecular function, and biological process. The gene product encoded by a gene performs an elementary low-level activity. This activity is described by ontologies as (molecular function). This activity occurs in a specific location of the cell. This location is modeled as a (cellular component). The elementary activities cooperate to perform a more general and larger scale activity described as a (biological process). The cellular component is the most visible aspect of the proposed ontology. Indeed, SARSMutOnto describes the genome of the SARS-CoV-2 virus as well as its different component genes. The activities are not the focus of this study. According to its type, each of the 15 genes that make up the genome is presented as an individual of one of the three classes designating the ‘structural_gene’, ‘non-structural_gene’, and ‘accessory_gene’ gene types, as shown in Figure 5.

Lineages are represented by classes inheriting directly or indirectly from the superclass ‘lineage’. The first two lineages A and B are represented by the ‘A’ and ‘B’ classes, which inherit directly from the ‘lineage’ class. The other lineages are linked to each other and to ‘A’ and ‘B’ by hierarchical links. The class that represents a given lineage is a subclass of the class representing the ancestor of this lineage and is the superclass of all classes representing its descendants, which allows the hierarchical relationship (lineage/sub-lineage) between lineages to be maintained. A class representing a recombinant lineage inherits the ‘recombinant’ class and all classes representing its parent lineages. Each class representing a lineage has a corresponding individual that provides the information characterizing this lineage by ‘dataTypeProperty’ assertion. This information includes a brief lineage description, an alias if available, and the first appearance date, as well as all the mutations produced with respect to the first strain of the virus. Each mutation is represented by an individual of the ‘SNP’ class. It is linked to the lineage, in which it occurs by the assertion of the ObjectProperty ‘has_for lineage’, and to the affected gene by the assertion of the ObjectProperty ‘has_for gene’. The SARSMutOnto ontology consists of 2206 classes and 2886 individuals and is available via Bioportal, the repository of biomedical ontologies.

### 4.2. Lineage Description

The taxonomy of classes proposed by SARSMutOnto illustrates the hierarchy of all lineages. It allows us to represent the phylogenetic tree of the Pango lineages. Thus, for a given lineage, all ancestors and descendants of this tree can be obtained and, therefore, all mutations that led to the appearance of each lineage can be obtained too. For example, the Delta variant also called B.1.617.2, triggered the fourth wave of the pandemic. This lineage emerged as a result of a succession of mutations from the B lineage, one of the two earliest observed strains of the virus: First the emergence of the lineage B.1 with the following mutations: S(d614g), ORF1b(P314L), and ORF855s84l), then B.1.617 with the mutations S(L452R, D614G, P681R), ORF1B(P314L), ORF3a(S26L), ORF7a(V82A), ORF8(S84L), and N(R203M, D377Y). Finally, B.1.617.2, which underwent mutations S(T19R, E156G, del157/158, L452R, T478K, D614G, P681R, D950N), ORF1B(P314L, G662S, P1000L), ORF3a(S26L), M(I82T), ORF7a(V82A, T120I), ORF8(S84L, del119/120), and N(D63G, R203M, D377Y) (Table 1). For this example, as we can see in the SARSMutOnto segment in Figure 5, the ancestor hierarchy of B.1.617.2 is represented by classes. Each class is linked to its direct ancestor by the ‘is_a’ relationship. Mutations are represented by individuals of the ‘SNP’ class. These individuals are linked to the lineage by assertions of the ‘has_for_lineage’ objectProperty, and to the concerned gene by the assertions of the ‘has_for_gene’ objectProperty. The classes representing the recombinant lineages inherit directly from the ‘recombinant’ class, in addition to the classes representing their parents. Hence, all classes representing recombinant lineages are descendants of the ‘recombinant’ class.

### 4.3. Querying SARSMutOnto

The SARSMutOnto ontology allows us to easily find the list of mutations associated with each lineage. It can be used by biologists or virologists to extract different types of information about SARS-CoV-2 mutations, for example, the list of all ancestors of a variant, the list of mutations that have been located in a given gene for all variants combined, the list of all lineages with a given mutation, the list of variants with a name assigned by the WHO, etc. Hereafter, some examples of SPARQL queries interrogating the SARSMutOnto ontology performed with the SPARQL language, using the twinkle tool (http://ldodds.com/projects/twinkle/, accessed on 20 January 2023) are given. More examples are available in Appendix A.

#### 4.3.1. List of Lineage Mutations

The first example aims at extracting a given lineage, the list of mutations, as well as the genes concerned by these mutations. The query in Listing 1 allows the mutations of the B.1.617.2 variant and the genes where they occur to be extracted.

**Listing 1.** Query to extract the mutation list of B.1.617.2 variant.

PREFIX

 ns:<https://github.com/jbakkas/SARSMutOnto/

blob

/main/SARSMutOnto.owl#>


SELECT

 ?mutationName ?gene


FROM

 <https://raw.githubusercontent.com/jbakkas/SARSMutOnto/main/SARSMutOnto.owl>


WHERE

{

  ?mutation   a   ns:SNP.

  ?lineage    a   ns:B.1.617.2.

  ?mutation    ns:has_for_lineage  ?lineage.

  ?mutation  ns:has_for_gene      ?gene .

  ?mutation   ns:mutation_name   ?mutationName

}

ORDER BY DESC

 ( ? gene )


#### 4.3.2. List of Lineages with a Given Mutation

A mutation can endow the virus with a specific characteristic that can cause severe forms of disease or make it more resistant to human immunity or a vaccine. To more effectively reduce the spread of such mutations, scientists need a list of all variants or lineages that carry this mutation. The following example, Listing  2, is a SPARQL query to retrieve the list of lineages containing the ’N501Y’ mutation of the Omicron variant. sub-lineages with this mutation are more infectious and dangerous for patients with cancer  [39].

**Listing 2.** Query to extract lineages with a given mutation.

PREFIX

 ns:<https://github.com/jbakkas/SARSMutOnto/

blob

/main/SARSMutOnto.owl#>


SELECT

 ?lineageName


FROM

<https://raw.githubusercontent.com/jbakkas/SARSMutOnto/main/SARSMutOnto.owl>


WHERE

{

  ?snp   a   ns:SNP.

  ?snp   ns:has_for_lineage   ?lineage.

  ?lineage  ns:label   ?lineageName.

  Filter (? snp=ns : N501Y)

}

ORDER BY

 ?lineageName


#### 4.3.3. List of Gene Mutations

Further useful information for biologists is the list of mutations that have occurred in a given gene since the virus first appeared, in particular, the list of mutations that have occurred in the spike protein S gene encoding the surface protein. The query in Listing  3 allows the extraction of the list of all mutations that have occurred in the spike protein gene S, all variants combined.

**Listing 3.** Query to extract the list of all mutations occurring in the spike (S) protein.

PREFIX

 owl:<http://www.w3.org/2002/07/owl#>


PREFIX

 ns:<https://github.com/jbakkas/SARSMutOnto/blob/main/SARSMutOnto.owl#>


SELECT

 ?mutationName ?gene


FROM

 <https://raw.githubusercontent.com/jbakkas/SARSMutOnto/main/SARSMutOnto.owl>


WHERE

{

  ?mutation  a            ns:SNP.

  ?mutation  a            owl:NamedIndividual.

  ?mutation  ns:has_for_gene    ns:S.

  ?mutation  ns:mutation_name   ?mutationName.

}


#### 4.3.4. List of Recombinant Lineages

The wide circulation of various variants of the virus generates recombinant lineages. Using a simple SPARQL query, we can list all Pango lineages resulting from a recombinant mutation of SARS-CoV-2. The query in Listing 4 returns a list of all Pango recombinant lineages with each lineage’s parents.

**Listing 4.** Query to extract the list of all Pango reconbinant lineages with their parents.PREFIX ns:<https://github.com/jbakkas/SARSMutOnto/blob/main/SARSMutOnto.owl#> PREFIX rdfs:<http://www.w3.org/2000/01/rdf-schema#> PREFIX rdf:<http://www.w3.org/2000/01/rdf-schema#> PREFIX owl:<http://www.w3.org/2002/07/owl#> SELECT ?l ?c FROM <https://raw.githubusercontent.com/jbakkas/SARSMutOnto/main/SARSMutOnto.owl> WHERE{   ?l rdf:subClassOf  ns:recombinant.   ?l rdf:subClassOf  ?c.   Filter (?c!=ns:recombinant) }

## 5. Conclusions

This paper contributes extensively to the design and implementation of a novel ontology called SARSMutOnto. This ontology is designed to describe the SARS-CoV-2 lineages and mutations. It provides the concepts and semantic entities necessary for studies and research that deal with mutations and variants of the SARS-CoV-2 virus. It is intended primarily for use by semantic interoperability approaches or for text mining in the SARS-CoV-2 domain. As this virus is in continuous mutation, lineages and even variants will constantly appear; the updated release of the ontology can be generated thanks to the aforementioned SARSMutOnto generator tool. The updated ontology release can be found at the Bioportal portal. Future research in this area will use machine learning techniques to predict possible future mutations of the SARS-CoV-2 virus based on the SARSMutOnto ontology. As part of our ongoing effort to harmonize ontologies, we will keep working to bring together different COVID-19-related ontologies. To manage the description of coronaviral variations, we will keep updating our ontology. Additionally, we will explore and design more applications that use this ontology.

## Figures and Tables

**Table 1:** Mutations affecting B.1.617.2 lineage   [38]. ORF: open reading frames gene.

Gene	Amino Acid
Spike protein gene	T19R
Spike protein gene	E156G
Spike protein gene	del157/158
Spike protein gene	L452R
Spike protein gene	T1T478K9R
Spike protein gene	D614G
Spike protein gene	P681R
Spike protein gene	D950N
Nucleocapsid gene	D63G
Nucleocapsid gene	R203M
Nucleocapsid gene	D377Y
ORF7a	V82A
ORF8a	S84L
ORF8a	del119/120
ORF1b	P314L
ORF1b	P1000L
ORF1b	G662S

## Data Availability

The datasets generated during and/or analysed during the current study are publicly available at https://bioportal.bioontology.org/ontologies/SARSMUTONTO/?p=summary.

## Appendix A. Additional Queries

This appendix presents some examples of SPARQL queries tested on the SARSMutOnto ontology. These queries are executed using the twinkle tool available via this link (http://ldodds.com/projects/twinkle/).

### Appendix A.1. Query 1

List of all lineages with a description and date of appearance for each lineage.

**Listing A1.** List of all lineages.
PREFIX ns:<https://github.com/jbakkas/SARSMutOnto/blob/main/SARSMutOnto.owl#> PREFIX owl:<http://www.w3.org/2002/07/owl#> prefix rdfs: <http://www.w3.org/2000/01/rdf-schema#>SELECT ?lineageName ?date ?description FROM <https://raw.githubusercontent.com/jbakkas/SARSMutOnto/main/SARSMutOnto.owl> WHERE{ ?lineage a owl:NamedIndividual. ?lineage ns:label ?lineageName ?lineage ns:appeared_on ?date. ?lineage ns:has_for_description ?description. }order by (?lineageName)

### Appendix A.2. Query 2

Date of appearance of a given lineage: the date of appearance of the Delta variant (B.1.617.2) for example.

**Listing A2.** The appearance date of a Delta variant (B.1.617.2).
PREFIX ns:<https://github.com/jbakkas/SARSMutOnto/ blob /main/SARSMutOnto.owl#> PREFIX owl:<http://www.w3.org/2002/07/owl#> prefix rdfs: <http://www.w3.org/2000/01/rdf-schema#>SELECT ?lineageName ?date FROM <https://raw.githubusercontent.com/jbakkas/SARSMutOnto/main/SARSMutOnto.owl> WHERE{ ?lineageI a owl:NamedIndividual. ?lineageI ns:label ?lineageName. ?lineageI ns:appeared_on ?date. FILTER (?lineageName=‘‘B.1.617.2’ ’) }

### Appendix A.3. Query 3

Lineages with OMS-assigned names. These are generally the most common variants.

**Listing A3.** List of lineages with WHO-assigned names.
PREFIX ns:<https://github.com/jbakkas/SARSMutOnto/blob/main/SARSMutOnto.owl#> PREFIX owl:<http://www.w3.org/2002/07/owl#> prefix rdfs: <http://www.w3.org/2000/01/rdf-schema#>SELECT ?lineageName ?WHO_namedate FROM <https://raw.githubusercontent.com/jbakkas/SARSMutOnto/main/SARSMutOnto.owl> WHERE{ ?lineage a owl:NamedIndividual. ?lineage ns:label ?lineageName. ?lineage ns:has_for_WHO_name ?WHO_name . FILTER(?WHO_name!=’ ’) }

### Appendix A.4. Query 4

Names of all sub-lineages of a given lineage. For example, the sub-lineages of the lineage (B.1.1.529 ) which represents the Omicron variant.

**Listing A4.** Sub-lineages of the lineage (B.1.1.529).
PREFIX ns:<https://github.com/jbakkas/SARSMutOnto/blob/main/SARSMutOnto.owl#> PREFIX owl:<http://www.w3.org/2002/07/owl#> prefix rdfs: <http://www.w3.org/2000/01/rdf-schema#>SELECT ?name FROM <https://raw.githubusercontent.com/jbakkas/SARSMutOnto/main/SARSMutOnto.owl> WHERE{ ?subLineage rdfs:subClassOf ns:B.1.1.529. ?ind a owl:NamedIndividual. ?ind a ?subLineage. ?ind ns:label ?name }
